# Clinical signs and symptoms cannot reliably predict *Plasmodium falciparum* malaria infection in pregnant women living in an area of high seasonal transmission

**DOI:** 10.1186/1475-2875-12-464

**Published:** 2013-12-27

**Authors:** Marc C Tahita, Halidou Tinto, Joris Menten, Jean-Bosco Ouedraogo, Robert T Guiguemde, Jean Pierre van Geertruyden, Annette Erhart, Umberto D’Alessandro

**Affiliations:** 1 Institut de Recherche en Sciences de la Santé/Direction Régionale de l’Ouest, 01 BP 545 Bobo-Dioulasso, Burkina Faso; 2 Laboratory of Parasitology and Entomology, Centre Muraz, Bobo Dioulasso, 01 BP 390 Bobo-Dioulasso, Burkina Faso; 3 Department of Public Health, Institute of Tropical Medicine, Nationalstraat155, B-2000 Antwerpen, Belgium; 4 International Health Unit, University of Antwerp, Antwerp, Belgium; 5 Disease Control and Elimination Theme, Medical Research Council Unit, Fajara, The Gambia

**Keywords:** Malaria, Pregnancy, Signs and symptoms

## Abstract

**Background:**

Malaria in pregnancy is a major public health problem in endemic countries. Though the signs and symptoms of malaria among pregnant women have been already described, clinical presentation may vary according to intensity of transmission and local perceptions. Therefore, determining common signs and symptoms among pregnant women with a malaria infection may be extremely useful to identify those in need of further investigation by rapid diagnostic test or microscopy.

**Methods:**

Six hundred pregnant women attending the maternity clinic of Nanoro District Hospital, Burkina Faso were recruited, 200 with suspected clinical malaria and 400 as controls. Cases were matched with controls by gestational age and parity. Signs and symptoms were collected and a blood sample taken for rapid diagnostic test, microscopy and haemoglobin measurement. A multivariate model was used to assess the predictive value of signs and symptoms for malaria infection.

**Results:**

The overall prevalence of malaria was 42.6% (256/600) while anaemia was found in 60.8% (365/600) of the women. Nearly half (49%) of the cases and 39.5% of the controls had a malaria infection (p = 0.03). The most common signs and symptoms among the cases were fever (36%,72/200), history of fever (29%,58/200) and headache (52%,104/200). The positive predictive value for fever was 53% (95% CI:41–64), history of fever 58% (95% CI:37–63) and headache 51% (95% CI:41–61).

**Conclusion:**

Signs and symptoms suggestive of malaria are frequent among pregnant women living in areas of intense transmission. Common malaria symptoms are not strong predictors of infection. For a better management of malaria in pregnancy, active screening to detect and treat malaria infection early should be performed on all pregnant women attending a health facility.

## Background

Malaria in pregnancy (MiP) is a major public health problem in endemic countries where 31 million pregnancies occur annually, resulting in approximately 23 million live births [1]. Where malaria transmission is moderate to high and stable, the most common parasite species is *Plasmodium falciparum* and MiP is predominantly asymptomatic and yet a major cause of maternal anaemia and low birth weight (LBW), the latter increasing the risk of infant death. Successful control of MiP may prevent 75,000-200,000 infant deaths every year [2]. In areas of low transmission, women by the time they become pregnant have acquired little immunity to malaria and so infections are often symptomatic and more likely to become severe, resulting in maternal and foetal deaths [2,3]. To prevent the consequences of MiP, the World Health Organization (WHO) recommends intermittent preventive treatment during pregnancy (IPTp), adequate management of clinical malaria, administering of supplements such as iron and folic acid, and the use of insecticide-treated nets [4].

The current strategy for clinical case management of malaria in pregnant women is based on the result of rapid diagnostic tests (RDT) or microscopy if available. Though the signs and symptoms of malaria among pregnant women have been already described in few settings, clinical presentation may vary according to intensity of transmission and local perceptions. Therefore, determining common signs and symptoms among pregnant women with a malaria infection may be extremely useful to identify those in need of further investigation by rapid diagnostic test or microscopy [5-7]. Furthermore, there is little information on the association between peripheral parasitaemia and the presence of signs and symptoms of malaria during pregnancy. This study aimed to document the clinical presentation of malaria among pregnant women and assess their predictive value.

## Methods

### Study area

The study was carried out at the Clinical Research Unit of Nanoro (CRUN), situated in the centre of Burkina Faso, 85 km from Ouagadougou, the capital city. Nanoro district is one of the five districts of the Centre-West health region. The main local language is Mooré, though French is the official language. The literacy rate is low for both men and women (about 23%) and there is a high migration flux among the youngsters toward the capital city and/or the border countries.

Malaria transmission is high with the entomological inoculation rate (EIR) in 2009 estimated at 50–60 infective bites/person/year (Diabate, pers comm). The more common vectors are *Anopheles gambiae sensu stricto*, *Anopheles funestus* and *Anopheles arabiensis*, and malaria is one of the most common reasons for attending a health facility [8]. *Plasmodium falciparum* is the predominant malaria parasite. Malaria transmission is seasonal, during the months of August-December, and overlaps with the rainy season (July-October). The peak for malaria cases usually occurs in September-October.

Since February 2009, the CRUN has implemented a demographic surveillance system (DSS) involving about 54,000 individuals. Some important information such as socio-economic status, e g, characteristics of households, living conditions, health conditions, are collected through the DSS on a regular basis.

The national anti-malarial drug policy was changed in 2005 from chloroquine to either amodiaquine-artesunate (AQ-AS) or artemether-lumefantrine (AL) as first-line treatments. Pregnant women received an insecticide-treated net (ITN) free of charge as soon as they complete two prenatal consultations or after delivery, while children receive theirs at each vaccination campaign. Nevertheless, a campaign for the promotion of insecticide-treated materials (ITM) targeting pregnant women and children under five years has been implemented since 2010 and nowadays an ITN is given to any pregnant woman attending for the first time during pregnancy an health facility.

### Study design and procedures

The study protocol was approved by the Institutional Ethic Committee of Centre Muraz (registration no. 005-2010/CE-CM). All pregnant women attending either the routine antenatal care (ANC) or the outpatient clinic were asked to participate in the study. During the visit and after having obtained a written informed consent, women were divided in cases or controls. Cases had at least one of the following signs and symptoms: temperature ≥37.5°C (measured by electronic thermometer) or history of fever in the previous 48 hours, headache, pallor, arthromyalgia, convulsions, vomiting, dizziness, malaise, fatigue, enlarged liver or enlarged spleen. Controls had none of them. For each case, two controls, matched by parity (0, 1–3, ≥4), gestational age (measured by fundal height) and seasonality (recruited within one month from the corresponding case). In order to capture possible seasonal variations in the clinical presentation, the study was conducted during both the low and high malaria transmission season.

Capillary blood was collected for an HRP-2 RDT, haemoglobin and blood slide. Women with a positive RDT were treated with AQ-AS (standard adult dose) if they were in the second and third trimester of pregnancy while quinine was given for seven days to those in the first trimester (8 mg quinine base/kg every eight hours). Severe malaria cases were hospitalized and treated with parenteral quinine. Anaemia was treated according to the national guidelines with oral ferrous sulphate (200 mg) and folic acid (0.25 mg) daily for one month. All patients with malaria parasites and anaemia were promptly and adequately treated free of charge. Project staff was available 24 hours a day to identify women and to ensure adequate documentation and clinical management.

### Laboratory methods

RDTs were carried out and results read according to the manufacturer’s procedures. All slides were read twice by two independent readers. Parasite density was estimated by counting the number of asexual parasites per 200 leukocytes in the thick blood film and assuming white blood cells (WBC) count of 8,000 parasites/μl. In case of discrepancy between the two readers (positive/negative, more than two-fold difference for parasite densities ≥400/μl, or more than the log_10_ for those <400/μl) [9], a third independent reading was done. The latter was taken as the final result. Haemoglobin was measured using a portable spectrophotometer (Hemocue®, Angelholm, Sweden) and controls were run daily to ensure the quality of the results.

### Sample size calculation

The sample size was calculated using the power calculation module for a two-sample comparison in order to detect with a two-sided significance level of 5% and 90% power important predictors of malaria infection in pregnant women. As there are no data available for the study area, the estimation was based on a study conducted in Mozambique [5]. It was assumed that 67% women would report history of fever and that among them 33% would be infected with malaria, while the prevalence of infection among the other women would be 20%. Following these parameters, six hundred pregnant women (200 cases and 400 controls) were needed. With an expected malaria prevalence of 27%, ie, 162 infected women, 16 possible predictors could be assessed, on the assumption that each of them would need at least ten events.

### Statistical methods and definitions

Double data entry, validation and cleaning were done using Epidata 3.1, and statistical analysis was performed with STATA.10 (STATA Corporation, College Station, TX, USA). Participants were categorized first as (i) cases and (ii) controls. The frequencies of signs and symptoms were compared between these groups. Odds ratio (OR) estimates and their confidence intervals (CI) were estimated in log binomial regression analyses. A P-value of ≤0.05 was considered statistically significant. The anaemia was classified as mild (Hb <11 g/dl), moderate (Hb 7–9 g/dl) and severe (Hb <7 g/dl). Parasite density was log transformed for the comparisons of mean parasite densities between cases and controls.

## Results

Most recruited women were either in the second (34.5%, 207/600) or third (60.5%, 363/600) trimester of pregnancy, and the mean age was 27.6 years (95% CI:27.1-28.0). More than half of them had already had one to three pregnancies and most of them were married (Table 1). The large majority of them worked at home (99%, 593/600) and could not read (92.1%, 553/600). Most women slept under a bed net (86.5%%). Baseline characteristics were similar between cases and controls (Table 1).

**Table 1:** Baseline characteristics of the recruited pregnant women by study group (%)

		**Cases**	**Controls**	**Total**
		**N = 200**	**N = 400**	**N = 600**
Age group	<20 years	18 (9.0)	23 (5.8)	41 (6.8)
	20-34 years	160 (80.0)	328 (82.0)	488 (81.3)
	≥35 years	22 (11.0)	49 (12.2)	71 (11.8)
Trimester ǂ	1st	10 (5.00	20 (5.0)	30 (5.0)
	2nd	84 (42.0)	123 (30.8)	207 (34.5)
	3rd	106 (53.00	257 (64.2)	363 (60.5)
Parity	0	17 (8.5)	34 (8.5)	51 (8.5)
	1-3	118 (59.0)	237 (59.3)	355 (59.2)
	≥4	65 (32.5)	129 (32.2)	194 (32.3)
Transmission season^Φ^	No	47 (23.5)	8 (2.0)	55 (9.2)
	Yes	153 (76.5)	392 (98.0)	545 (90.8)
Sleep under bed net		155 (77.5)	364 (91.0)	519 (86.5)
Malaria infection		98 (49.0)	158 (39.5)	256 (42. 7)

ǂ1^st^ trimester: 0–12 weeks; 2^nd^ trimester: 13–24 weeks; 3^rd^ trimester: ≥25.

ΦTransmission season: August to December, no transmission season: June to July.

**Figure 1:**
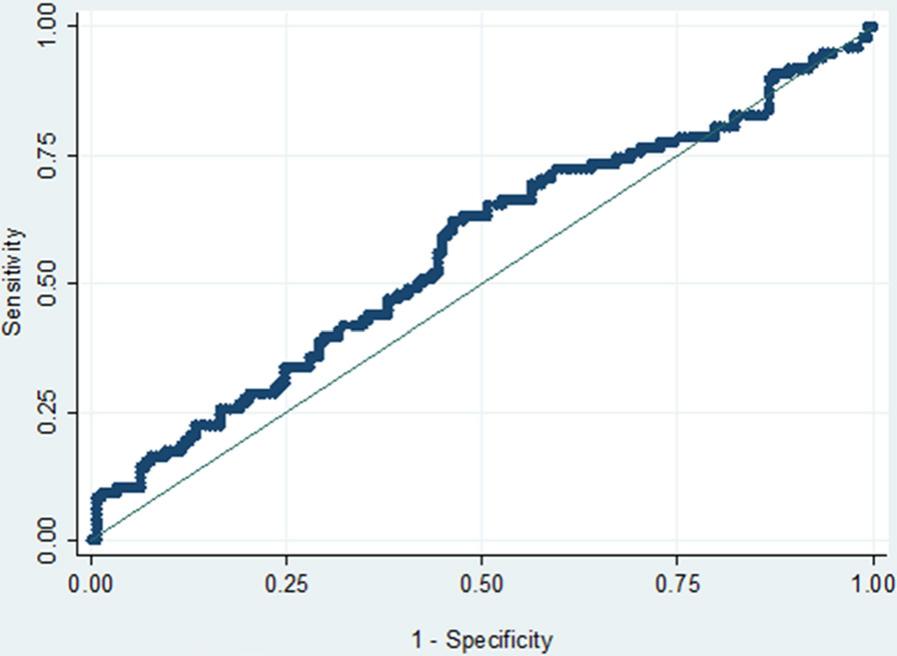
ROC curve assessing the parasite density discriminating a case from a control (case threshold).

The prevalence of malaria infection using the RDT was 60% among the cases and 47.8% among the controls. When using microscopy, the prevalence of malaria infection was 49.0% among cases and 39.5% among controls (p = 0.03). The latter would not have been treated in case of clinical diagnosis. The mean (geometric) parasite density among infected women was not significantly different among cases (3.0/μl, 95% CI: 2.9-3.2) and controls (2.8/μl, 95% CI:2.7-3.0) (p = 0.06). This is clearly illustrated by the Receiver Operating Characteristics (ROC) curve, with only 56% under the curve and no clear-cut off point distinguishing a case from a control (Figure 1). More than half of infected women (56.3%, 144/256) had a parasite density <1,000/μl. Malaria infection was strongly associated with anaemia (OR:1.96, 95% CI:1.40-2.74; p < 0.01), with more than half of all malaria-infected women (68.7%, 176/256) being anaemic. Among symptomatic cases, age was significantly related to malaria infection, with women <20 years more at risk than those ≥35 years old (Table 2). Conversely, neither season gestational age or bed net use were significantly associated with malaria, though there was the tendency of having a lower risk of malaria with bed nets, in multigravidae and before the transmission season (Table 2).

**Table 2:** ***Plasmodium falciparum *** **infection by risk factors among symptomatic pregnant women**

	**Risk factors**	**n/N (%)**	**OR (95%CI)**	**P-value**
Age	<20 years	11/16 (69.0)	Ref	--
	20-34 years	79/158 (50.0)	0.45 (0.15-1.37)	0.16
	≥35 years	8/26 (31.0)	0.20 (0.05-0.78)	0.02
Transmission season	No	16/47 (34.0)	Ref	---
	Yes	82/153 (53.0)	1.59 (0.88-2.86)	0.12
Parity	Nulliparous	10/17 (59.0)	Ref	--
	1-3	59/100 (50.0)	0.70 (0.25-1.95)	0.49
	≥4	29/65 (45.0)	0.56 (0.19-1.66)	0.30
Gestational age	1st	3/10 (30.0)	Ref	---
	2nd	48/84 (57.1)	3.31 (0.75-12.86)	0.11
	3rd	47/106 (44.3)	1.85 (0.45-7.58)	0.38
Bed net use	Yes	20/50 (40.0)	Ref	---
	No	76/147(51.7)	1.60 (0.83-3.08)	0.15

**Figure 2:**
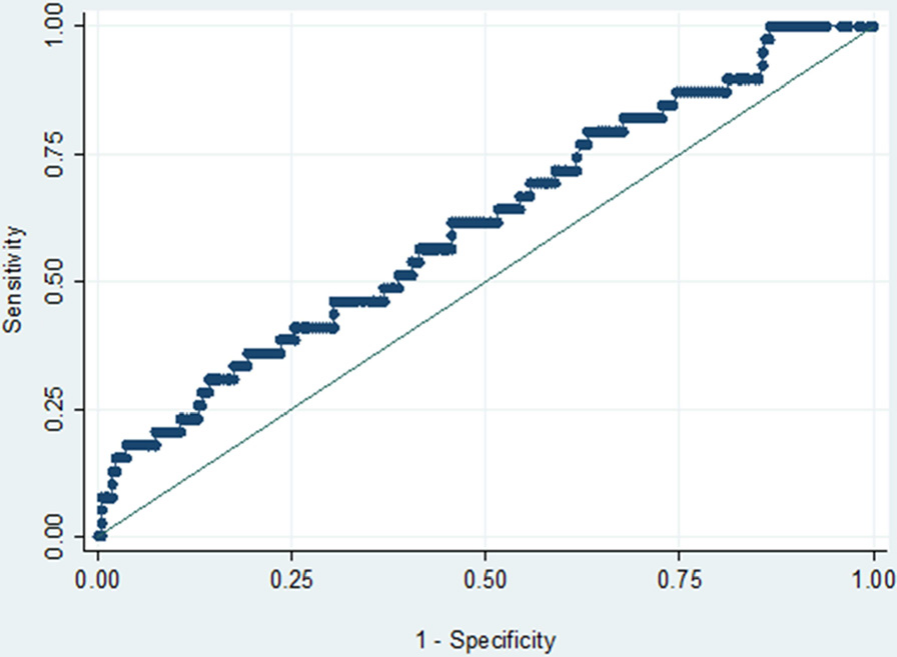
ROC curve assessing the parasite density discriminating women with or without fever (fever threshold).

Fever, history of fever, headache, and dizziness each had a positive predictive value (PPV) around 50%, while for all the others the PPV was well below (Table 3). The highest PPV were found when combining fever and dizziness (61.5%, 95%CI:35–82), and fever and vomiting (66.7%, 95%CI:20–93). If fever had been used to diagnose malaria, 47.2% of the febrile women would have been unnecessarily treated. Also 46.8% of the non-febrile women would have missed treatment for their malaria infection. When considering only fever as symptom, the ROC curve was flat, indicating that no fever “threshold” could be determined (Figure 2).

**Table 3:** **Positive predictive value (PPV) of clinical signs and symptom for *Plasmodium falciparum *** **infection among the 200 pregnant women recruited as cases**

**Signs/Symptoms**	**n**	**%**	**PPV (%)**	**(95%CI)**
Fever	38	19.0	53	(41–64)
History of fever	29	14.5	58	(37–63)
Pain in the joints	6	3.0	25	(6 – 44)
Headache	57	28.5	51	(41 – 61)
Dizziness	3	1.5	47	(41–64)
Vomiting	3	1.5	38	(37–63)
Convulsions	1	0.5	33	(6–79)
Malaise	7	3.5	30	(41 –61)

The occurrence of signs and symptoms was associated with higher parasite densities; in women complaining of headache, parasite density was significantly higher than in those without (6.4/μl *vs* 1.8/μl)(p = 0.003). Moreover, the mean (geometric) parasite density tended to be higher in women with vomiting (14,617.8/μl *vs* 2.7/μl; p = 0.05), fever (5.7/μl *vs* 2.2/μl) (p = 0.07), and complaining of malaise (53.5 *vs* 2.7; p = 0.07).

In the univariate analysis, presence of symptoms (OR = 1.47, 95%CI:1.05-2.07; p = 0.03), fever (OR = 1.76, 95%CI:0.99-3.11; p = 0.05) and headache (OR = 1.53, 95% CI:0.96-2.46; p = 0.07) were or tended to be associated with malaria infection. In the multivariate model, fever and history of fever (OR = 1.83, 95%CI:1.16-2.88; p = 0.009) also fever and history of fever and headache infection (OR = 1.63, 95%CI:1.13-2.37; p = 0.01) were significantly associated with malaria infection (Table 4).

**Table 4:** Comparison of the association between signs/symptoms and malaria infection in symptomatic pregnant women and their matched controls

		**n/N (%)**	**OR (95% CI)**	**P value**
Any symptom	No	158/400 (40)	Ref.	---
	Yes	95/200 (49)	1.47 (1.05-2.07)	0.03
Fever	No	56/144 (39)	Ref.	---
	Yes	38/72 (53)	1.76 (0.99-3.11)	0.05
History of fever	No	39/110 (35)	Ref.	
	Yes	27/55 (49)	1.76 (0.91-3.39)	0.09
Joint pain	No	14/48 (29)	1.23 (0.40 - 3.76)	0.71
	Yes	6/24 (25)	ref	---
Headache	No	84/208 (40)	Ref.	
	Yes	53/104 (51)	1.53 (0.96-2.46)	0.08
Dizziness	No	39/76 (51)	1.17 (0.53-2.55)	0.69
	Yes	18/38 (47)	Ref	---
Vomiting	No	9/16 (56)	2.14 (0.37-12.19)	0.39
	Yes	3/8(37)	ref	
Convulsions	No	0 (0)	-	
	Yes	1/3 (33)	-	
Malaise	No	17/48 (35)	1.33 (0.46-3.84)	0.60
	Yes	7/24 (29)	ref	
Fever and	No	84/230 (37)	Ref.	
history of fever	Yes	59/115 (51)	1.83 (1.16, 2.88)	0.01
Fever and	No	131/340 (39)	Ref.	
history of fever and headache	Yes	86/170 (51)	1.63 (1.13, 2.37)	0.01

## Discussion

In this study, malaria infection was extremely common among pregnant women, regardless of signs and symptoms. Signs and symptoms were not discriminative for malaria infections. In peripheral health facilities without access to parasitological diagnosis by either RDT or microscopy, health staff may have unnecessarily treated half of the women with signs and symptoms suggestive of malaria, while a substantial proportion of infected pregnant women would have gone undetected. Assuming diagnostic tests were available, the recommendation of performing them only on suspected cases would have still missed several infected women. This is particularly worrying for those in the first trimester of pregnancy as they do not receive the sulphadoxine-pyrimethamine (SP) for IPTp. Moreover, it has been repeatedly reported that coverage of IPTp with at least two administered doses of SP is still relatively low, despite women attending antenatal clinics several times during their pregnancy [10,11].

In endemic countries, assuming that any pregnant woman attending an antenatal clinic is infected with malaria, regardless of symptoms, seems to be the right approach. This means that pregnant women should be either systematically screened for malaria infection and treated if positive or given SP at any time they visit a health centre, provided this is done at least one month apart. The latter has been recently recommended by the WHO Malaria Policy Advisory Committee and Secretariat [12] though it has not been implemented widely yet. For women in the first trimester of pregnancy systematic screening is probably the best approach as they cannot be given SP as IPTp. Active detection of malaria infection among pregnant women can have a major impact on miscarriage and pre-term delivery [13,14] and maternal mortality. Indeed, at the north-western border of Thailand this strategy was associated with a six-fold decline in the overall maternal mortality ratio (MMR), with *P. falciparum* malaria related MMR falling from an estimated 1,000/100,000 live births prior to weekly screening to zero in 2005 [15].

The prevalence of malaria infection was significantly higher in women with symptoms suggestive of malaria but none of them or no combination of any of them had a very high PPV. This is confirmed by the weak association between malaria infection and any of the signs and symptoms, underlying the importance of parasitological diagnosis for any suspected case. The parasite density in pregnancy is not related to being a symptomatic patient and does not increase the probability that fever is related to malaria.

In places where transmission is high, malaria infection during pregnancy is often asymptomatic [16,17]. In Mozambique, signs and symptoms suggestive of malaria were extremely common in pregnant women but malaria infection was confirmed in only a minority of them [5]. The large majority of infections detected in pregnant women was asymptomatic in Benin [6]. Nevertheless, in Ghana malaria-infected pregnant women were often symptomatic, with history of fever, headache, vomiting, malaise, and dizziness occurring significantly more frequently than in uninfected women [7]. This is surprising when considering that the study was carried out in an area of stable and intense transmission. Indeed, in most of the previous reports from areas of intense transmission, malaria infection in pregnancy was often asymptomatic and this is also confirmed by this study. All common signs and symptoms, taken alone or in combination, had low PPV, with the only exception of two combinations, ie, fever and dizziness, and fever and vomiting. Nevertheless, even though the PPV for these two combinations was above 60%, about 30-40% of women with these complaints would have been unnecessarily treated for malaria. Pregnant women displaying signs and symptoms suggestive of malaria but without confirmed malaria should also be screened for others infectious diseases.

Several known risk factors such as maternal age [18,19] and parity [20] were confirmed to be associated with malaria infection during pregnancy, although in some instances the difference was not statistically significant, probably due to the need for a larger sample size. In addition, the risk of malaria infection did not vary significantly by gestational age, though the prevalence was the highest in the second trimester, an observation reported in other studies in which the peak prevalence during pregnancy was at 13–16 weeks of gestation [21,22].

## Conclusion

Malaria-related signs and symptoms, e.g. fever or headache, although more frequent in pregnant women with a malaria infection, cannot be used to identify those in need of treatment as a large proportion of asymptomatic women can be infected. Any pregnant women attending a health facility should be tested regardless of symptoms for malaria infection, either by microscopy or RDT. This would be particularly useful for women in the first trimester of pregnancy as they cannot be administered SP as IPTp.

## Competing interests

The authors declare that they have no competing interests.

## Authors’ contributions

The study was conceived by UDA and this paper drafted by MCT and UDA. It was conducted by MCT and HT with substantial contributions from JM. Data analyses were conducted by MCT, and supervised by AE and JM. AE, JBO, RTG, JPVG, HT and UDA participated in the overall running of the study, contributed to the interpretation of data, and gave critical review of the final draft. All authors read and approved the final version.

## References

[B1] Dellicour S Tatem AJ Guerra CA Snow RW ter Kuile FO Quantifying the number of pregnancies at risk of malaria in 2007: a demographic study PLoS Med 2010 12 1 10.1371/journal.pmed.1000221 PMC2811150 20126256

[B2] Rodriguez-Morales AJ Sanchez E Vargas M Piccolo C Colina R Arria M Franco-Paredes C Pregnancy outcomes associated with *Plasmodium vivax* malaria in Northeastern Venezuela Am J Trop Med Hyg 2006 12 755 757 16687675

[B3] Singh N Mehra RK Srivastava N Malaria during pregnancy and infancy, in an area of intense malaria transmission in central India Ann Trop Med Parasitol 2001 12 15 29 10.1080/00034980020035889 11235550

[B4] WHO/AFRO A strategic framework for malaria prevention and control during pregnancy in the African region 2004: AFR/MAL/04/01

[B5] Bardaji A Sigauque B Bruni L Romagosa C Sanz S Mabunda S Mandomando I Aponte J Sevene E Alonso PL Menendez C Clinical malaria in African pregnant women Malar J 2008 12 27 10.1186/1475-2875-7-27 18234078 PMC2267805

[B6] Huynh BT Fievet N Gbaguidi G Borgella S Mevo BG Massougbodji A Deloron P Cot M Malaria associated symptoms in pregnant women followed-up in Benin Malar J 2011 12 72 10.1186/1475-2875-10-72 21453493 PMC3076273

[B7] Tagbor H Bruce J Browne E Greenwood B Chandramohan D Malaria in pregnancy in an area of stable and intense transmission: is it asymptomatic? Trop Med Int Health 2008 12 1016 1021 10.1111/j.1365-3156.2008.02111.x 18631316

[B8] Ministère de la Santé (Burkina Faso) Statistiques sanitaires 1994 Ouagadougou:

[B9] Greenwood BM Armstrong JRM Comparison of two simple methods fordetermining malaria parasite density Trans R Soc Trop Med Hyg 1991 12 186 188 10.1016/0035-9203(91)90015-Q 1887466

[B10] Koen P Gies S Coulibaly SO Ky C Somda J Toomer E Ribera JM D’Alessandro U Bottlenecks for High coverage of intermittent preventive treatment in pregnancy: the case of adolescent pregnancies in rural Burkina Faso PLoS One 2010 12 8 10.1371/journal.pone.0012013 PMC2917368 20700460

[B11] Chima AO Kara H Obinna EO Low coverage of intermittent preventive treatment for malaria in pregnancy in Nigeria:demand-side influences Malar J 2012 12 82 10.1186/1475-2875-11-82 22443266 PMC3364889

[B12] World Health Organization Malaria Policy Advisory Committee and Secretariat Malaria Policy Advisory Committee to the WHO:conclusions and recommendations of September 2012 meeting Malar J 2012 12 424 23253143 10.1186/1475-2875-11-424 PMC3558335

[B13] Bardaji A Sigauque B Sanz S Maixenchs M Ordi J Aponte JJ Mabunda S Alonso PL Menendez C Impact of malaria at the end of pregnancy on infant mortality and morbidity J Infect Dis 2011 12 691 699 10.1093/infdis/jiq049 21199881 PMC3071276

[B14] Luxemburger C McGready R Kham A Morison L Cho T Chongsuphajaisiddhi T White NJ Nosten F Effects of malaria during pregnancy on infant mortality in an area of low malaria transmission Am J Epidemiol 2001 12 459 465 10.1093/aje/154.5.459 11532788

[B15] McGready R Boel M Rijken MJ Ashley EA Cho T Moo O Paw MK Pimanpanarak M Hkirijareon L Carrara VI Lwin KM Phyo AP Turner C Chu CS van Vugt M Price RN Luxemburger C ter Kuile FO Tan SO Proux S Singhasivanon P White NJ Nosten FH Effect of early detection and treatment on malaria related maternal mortality on the north-western border of Thailand 1986–2010 PLoS One 2012 12 7 10.1371/journal.pone.0040244 PMC3399834 22815732

[B16] Desai M ter Kuile FO Nosten F McGready R Asamoa K Brabin B Newman RD Epidemiology and burden of malaria in pregnancy Lancet Infect Dis 2007 12 93 104 10.1016/S1473-3099(07)70021-X 17251080

[B17] Nosten F Rogerson SJ Beeson JG McGready R Mutabingwa TK Brabin B Malaria in pregnancy and the endemicity spectrum: what can we learn? Trends Parasitol 2004 12 425 432 10.1016/j.pt.2004.06.007 15324733

[B18] Rogerson SJ van den Broek NR Chaluluka E Qongwane C Mhango CG Molyneux ME Malaria and anemia in antenatal women in Blantyre, Malawi: a twelve-month survey Am J Trop Med Hyg 2000 12 335 340 11037774 10.4269/ajtmh.2000.62.335

[B19] Dicko A Mantel C Aly Thera M Doumbia S Diallo M Diakete M Sagara I Doumbo O Risk factors for malaria infection and anemia for pregnantwomen in the Sahel area of Bandiagara, Mali Afr J Med Med Sci 2003 12 17 23 10.1016/j.actatropica.2003.07.001 14636978

[B20] Kyabayinze DJ Tibenderana JK Nassali M Tumwine LK Riches C Montague M Counihan H Hamade P Van Geertruyden JP Meek S Placental *Plasmodium falciparum* malaria infection: operational accuracy of HRP2 rapid diagnostic tests in a malaria endemic setting Malar J 2011 12 306 10.1186/1475-2875-10-306 22004666 PMC3206496

[B21] Brabin BJ An analysis of malaria in pregnancy in Africa Bull World Health Organ 1983 12 1005 1016 6370484 PMC2536236

[B22] Diagne N Rogier C Sokhna CS Tall A Fontenille D Roussilhon C Spiegel A Trape JF Increased susceptibility to malaria during the early postpartum period New Microbiol 2000 12 598 603 10.1056/NEJM200008313430901 10965006

